# Supplementary material for: An Effective Strategy for Template-Free Electrodeposition of Aluminum Nanowires with Highly Controllable Irregular Morphologies
Source: Nanomaterials (Basel). 2022 Apr 19;12(9):1390. doi: 10.3390/nano12091390 (PMC9105039; doi:10.3390/nano12091390)
Supplement: Supplementary file 1 [file nanomaterials-12-01390-s001.zip › nanomaterials-1629566-supplementary.pdf]

nanomaterials

MDPI

Supplementary materials

An Effective Strategy for Template-Free Electrodeposition of Aluminum Nanowires with Highly Controllable Irregular Morphologies

Heng Wang [1], Guo-Min Li [1], Bing Li [1,*] and Jing-Lin You [2,*]

[1] School of Mechanical and Power Engineering, East China University of Science and Technology, No.130 Mei long Road, Shanghai 200237, China; wh1wh2wh3wh@163.com (H.W.); liguominyumifei@163.com (G.-M.L.)

[2] State Key Laboratory of Advanced Special Steel, School of Materials Science and Engineering, Shanghai University, Shanghai 200444, China

* Correspondence: bingli@ecust.edu.cn (B.L.); jlyou@staff.shu.edu.cn (J.-L.Y.)

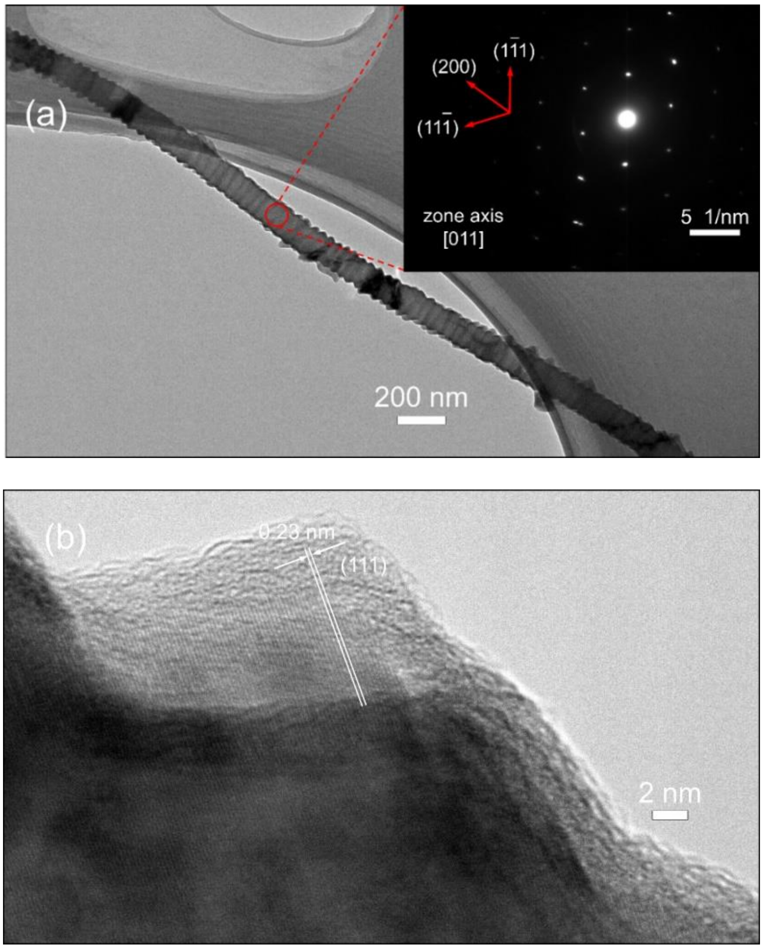

Figure S1. SAED pattern (inset in (**a**)) and HRTEM image (**b**) of the bellows-like Al nanowire electrodeposition at −1.0 V for 120 s at 25 °C with a 2 mm thick insulation ring (inner diameter was 6 mm).

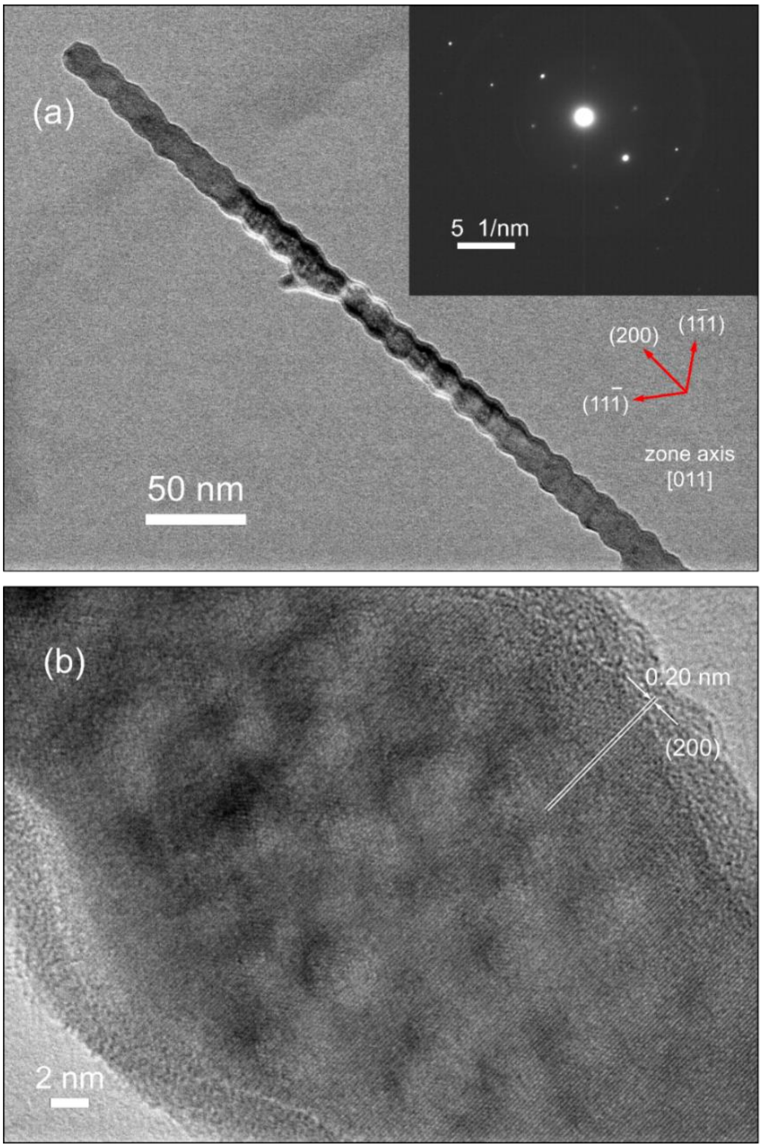

Figure S2. SAED pattern (inset in (**a**)) and HRTEM image (**b**) of the bead-chain-like Al nanowire electrodeposition at −1.2 V for 120 s at 25 °C with a 2 mm thick insulation ring (inner diameter was 6 mm).

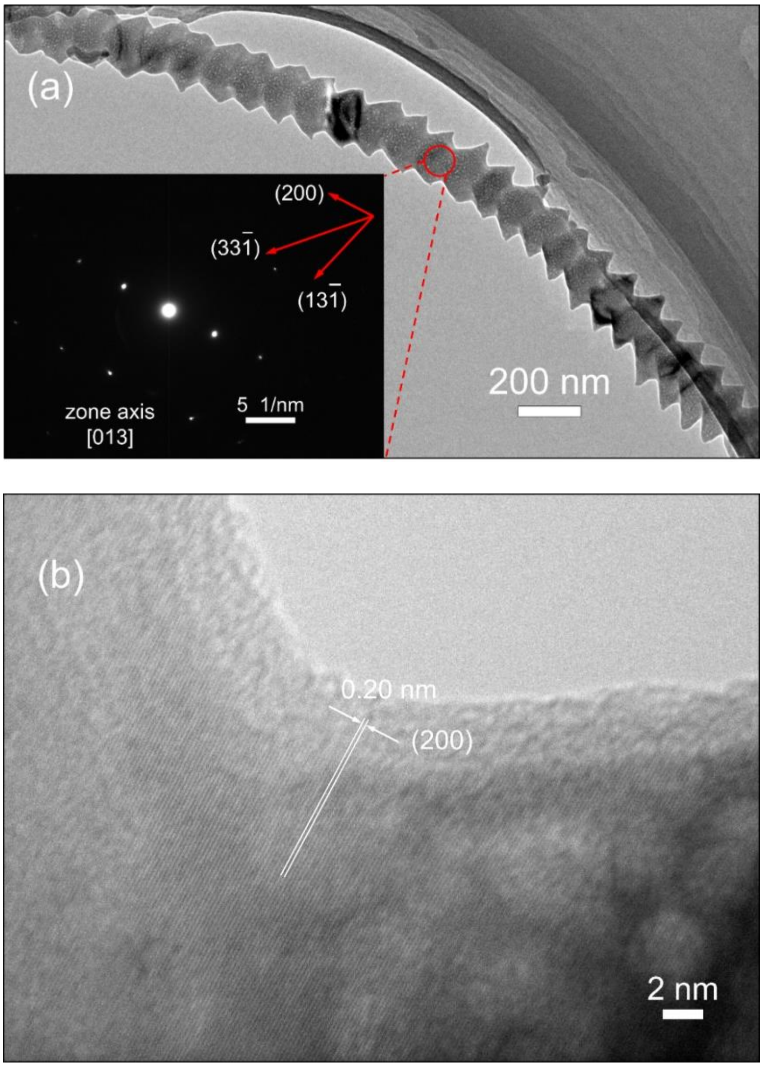

Figure S3. Selected area electron diffraction (SAED) pattern (inset in (**a**)) and HRTEM image (**b**) of the spine-like Al nanowire electrodeposition at −1.4 V for 120 s at 25 °C with a 2 mm thick insulation ring (inner diameter was 6 mm).
